# Thiosulfoxide (Sulfane) Sulfur: New Chemistry and New Regulatory Roles in Biology

**DOI:** 10.3390/molecules190812789

**Published:** 2014-08-21

**Authors:** John I. Toohey, Arthur J. L. Cooper

**Affiliations:** 1 Cytoregulation Research, Elgin, ON K0G1E0, Canada; 2 Department of Biochemistry and Molecular Biology, New York Medical College, Valhalla, NY 10595, USA; E-Mail: arthur_cooper@nymc.edu

**Keywords:** cystamine, cystathionine γ-lyase (γ-cystathionase), garlic, glutathione persulfide, hydrogen sulfide, mercaptoethanol, perseleno selenium, persulfide, sulfane sulfur, thioglycerol

## Abstract

The understanding of sulfur bonding is undergoing change. Old theories on hypervalency of sulfur and the nature of the chalcogen-chalcogen bond are now questioned. At the same time, there is a rapidly expanding literature on the effects of sulfur in regulating biological systems. The two fields are inter-related because the new understanding of the thiosulfoxide bond helps to explain the newfound roles of sulfur in biology. This review examines the nature of thiosulfoxide (sulfane, S^0^) sulfur, the history of its regulatory role, its generation in biological systems, and its functions in cells. The functions include synthesis of cofactors (molybdenum cofactor, iron-sulfur clusters), sulfuration of tRNA, modulation of enzyme activities, and regulating the redox environment by several mechanisms (including the enhancement of the reductive capacity of glutathione). A brief review of the analogous form of selenium suggests that the toxicity of selenium may be due to over-reduction caused by the powerful reductive activity of glutathione perselenide.

## 1. Introduction: Sulfur Bonding

Some long-held theories on sulfur bonding have been called into question by the use modern physico-chemical technology and enhanced computing ability. The challenged theories include the theory of hypervalency of sulfur and the nature of the S-S bond in thiosulfoxides. In 1982, Kutney and Turnbull published a paper titled “Compounds Containing the S=S Bond” [1]; however, new physical and computational data suggest that the S=S (double) bond may not exist. The nature of sulfur bonding relevant to life processes is outlined briefly below.

**Figure 1:**
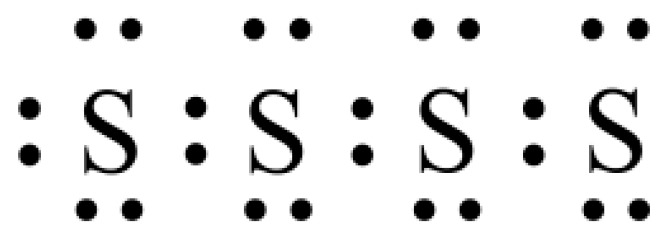
Bonding of elemental dulfur.

Chalcogen atoms (group 16 of the periodic table) have six electrons in the valence shell providing these atoms with special bonding possibilities. Atoms with an even number of valence electrons have the ability to catenate or bond together in series. Carbon (group 14), with four valence electrons, catenates in three dimensional lattices, but sulfur, with six valence electrons, forms chains of atoms bonded by 2-electron dative bonds (Figure 1). Branching can occur since a sulfur atom in a chain can donate electron pairs to more than one sulfur atom.

**Figure 2:**
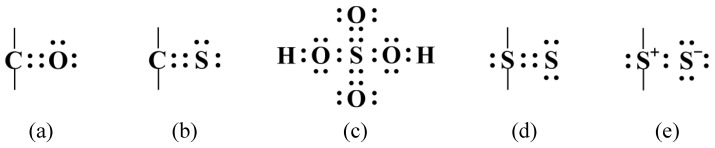
Sulfur bonding showing electron distribution

At high temperatures, very long chains form but, at lower temperatures, the chains cyclize in rings of eight atoms that can pack in several allotropic forms. Sulfur atoms have a high affinity for bonding to other chalcogens atoms, particularly oxygen as reflected in its ancient name “brimstone” (burning stone). The true nature of sulfur bonding is only now being revealed. Like oxygen, sulfur can form 4-electron dative bonds (compare below Figure 2a a ketone and Figure 2b a thione). For a long time, it was thought that sulfur, unlike oxygen, was able to accommodate more than eight Lewis electrons in its valence shell. This property, called hypervalency is exemplified in Figure 2c the traditional “text-book” representation of sulfuric acid in which the S atom has 12 valence electrons. However, the concept of hypervalency is now in question [2,3]. Recent studies of electron density mapping using synchrotron X-ray diffraction have shown that many chalcogen-chalcogen bonds previously considered to be 4-electron dative bonds are, in fact, 2-electron polar dative bonds [4]. The two alternative structures are shown in Figure 2d,e for thiosulfoxide. *Ab initio* calculations indicate that the thiosulfoxide bond is a polar 2-electron bond as shown in Figure 2e [5] and much weaker than the previously-assumed double bond shown in Figure 2d [6]. Therefore, thiosulfoxide sulfur is relatively reactive and this undoubtedly contributes to the regulatory functions of sulfane sulfur in biological systems as summarized in this review.

**Table 1:** Structure and nomenclature of sulfur compounds.

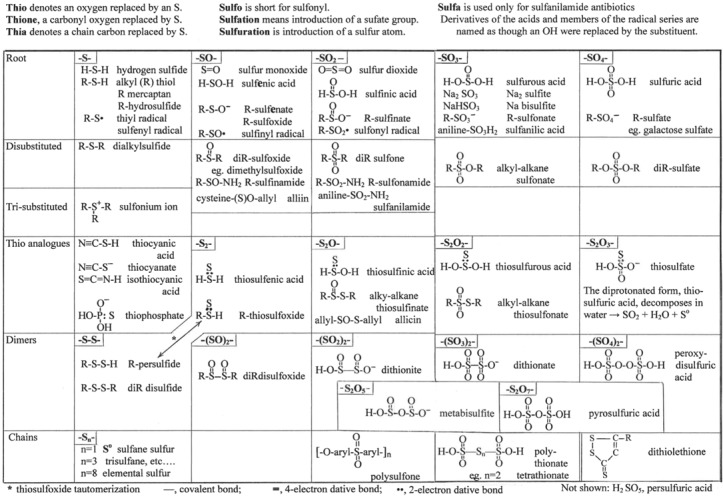

There are three systems of nomenclature for sulfur compounds based on the roots “sulf” (“sulph” in the UK), “mercapto”, and “thio”. Table 1 is a compilation of the structures and nomenclature of sulfur and sulfur-oxygen compounds. Some sulfur atoms in the structures are shown in the classical (4-electron) format but other bonds are shown as 2-electron bonds when the chemical and biological evidence supports this representation.

## 2. Sulfur in Biology

Because of the versatility of the sulfur atom and its prevalence in the primordial environment, it is not surprising that sulfur evolved to fill many structural, catalytic, and regulatory roles in biology. Sulfur is life-supporting in the following processes:

- Elemental sulfur reduction to H_2_S provides a source of energy in *Desulfuromonas* and archaea.
- H_2_S oxidation to elemental sulfur provides a source of energy in *Beggiatoa*.
- H_2_S or S^0^ oxidation to sulfate provides a source of energy in *Thiobacillus* and archaea.
- Sulfate or sulfite reduction to H_2_S provides a source of oxygen for *Desulfovibrio*, archaea.
- H_2_S splitting during photosynthesis provides a source of hydrogen atoms in purple and green sulfur bacteria.

Covalently-bonded sulfur, in a wide range of oxidation states, is a determinant of structure and function in many biological systems. Cysteine and methionine are primary structural elements of proteins and the sulfur of cysteine is an important determinant of the tertiary structure of proteins. The sulfhydryl group of glutathione is a major determinant of redox status in tissues. The sulfhydryl group on proteins is involved in regulating the activity of the proteins both by disulfide bond formation and by persulfuration. It is thought that reversible oxidation of SH groups to the sulfenyl form in regulatory proteins is a signaling mechanism [7] and that sulfuration of transfer RNA is a mechanism for controlling translation [8]. The sulfonyl group, -SO_3_^−^, provides detergent properties to taurine (2-aminoethane sulfonic acid), the major conjugant for the excretion of cholesterol-derived products in bile. Sulfate occurs as esters of numerous hydroxy compounds: carbohydrates, glycosylaminoglycans (e.g., heparin, chondroitin), lipids (such as cholesterol and sulfatides), proteins (hydroxyl groups of serine, tyrosine and threonine), and hormones (thyroxin). Sulfur is a key component in six major cofactors in mammals (iron-sulfur clusters, coenzyme A, lipoic acid, thiamine pyrophosphate, molybdenum cofactor, and biotin) and two additional cofactors in bacteria and archaea (coenzyme M and coenzyme B). The molybdenum cofactor (MoCo) functions in sulfite oxidase, xanthine oxidoreductase, and aldehyde oxidase in humans and in other enzymes in microorganisms and plants [9]. In MoCo, the pterin platform has two sulfur atoms that bind the Mo atom and, in aldehyde oxidase, there is a third sulfane sulfur atom terminally bonded to the Mo atom; all three sulfur atoms originate as S^0^ extracted from cysteine by pyridoxal 5'-phosphate (PLP)-containing cysteine desulfurases [9]. In the tungsten-containing enzymes of hyperthermophilic archaea, the Mo is replaced by its congener, W, on the two sulfur atoms of the same pterin platform [10].

Sulfane sulfur, which is sulfur in the thiosulfoxide form (represented as S^0^), has been found to have remarkable regulatory functions in biological systems. The following review briefly outlines the unveiling of these functions of sulfane sulfur, its unique nature, and its biogeneration.

## 3. Sulfur as a Regulatory Agent

**Figure 3:**
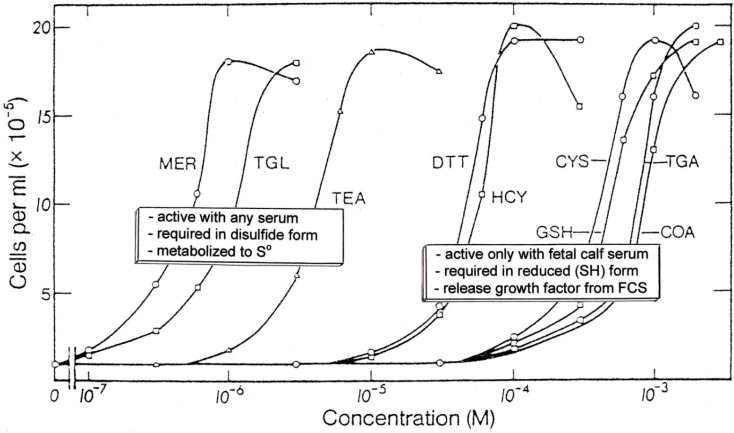
Growth response of P388 leukemia cells *in vitro* to various sulfur compounds. Cells were cultured in MEM in the presence of varied concentrations of the compounds: MER, 2-mercaptoethanol; TGL, thioglycerol; TEA, thioethanolamine; DTT, dithiothreitol; HCY, homocysteine; CYS, cysteine; GSH, glutathione; TGA, thioglycolic acid; COA, coenzyme A. (adapted from [16]).

Interest in sulfur as a regulatory agent began more than 40 years ago in studies with immune cell systems cultured *in vitro*. In 1970, Fanger and colleagues showed that cysteine, glutathione, or sulfite ion at mM concentrations in the presence of 20% fetal calf serum markedly enhanced the response of lymphocytes to transforming agents [11]. In 1972, Click and colleagues reported that 2-mercapto- ethanol (MER) at micromolar concentrations caused a 2- to 3-fold stimulation of antibody production [12] and T cell proliferation [13]. This finding was soon expanded to other immune systems and other sulfur compounds such as α-thioglycerol (TGL) [14]. The next breakthrough was in 1973 when Broome and Jeng reported that thiols or their disulfides permitted *in vitro* proliferation of murine cancer cell lines previously not culturable *in vitro* but carried in live mice [15]. In 1975, one of the present authors (JT) confirmed this growth factor effect with several members of a bank of murine cell lines and showed that the sulfur compounds fall into two categories [16]. As shown in Figure 3, three xenobiotic sulfur compounds, MER, TGL, and TEA (cysteamine, 2-mercapto-1-aminoethane, thioethanolamine) stimulate growth under the following conditions: (a) at μM concentrations; (b) only in the oxidized (disulfide) form [17]; and (c) with any serum (or bovine serum albumin) replacing fetal calf serum. 

Compounds in the second group (cysteine, glutathione, homocysteine, coenzyme A, thioglycolic acid, and dithiothreitol) are active: (a) only in the reduced (thiol) form, (b) at high (mM) concentrations, and (c) only in the presence of fetal calf serum. Sera other than fetal calf serum are ineffective with the second group [18]. Cystine is active at 1 mM in the presence of a pyridoxal catalyst [17].

The conclusion from these findings is that disulfides in the first group generate a growth factor *de novo* while the compounds in the second group mobilize the growth factor from fetal calf serum. The mechanism common to the first group is the metabolic generation of a carbonyl group adjacent to the disulfide bond resulting in the labilization of one of the sulfur atoms and its release as sulfane sulfur [17] (Table 2). The catalysts effective in the cell cultures were found to be alcohol dehydrogenase for the disulfides of mercaptoethanol and thioglycerol, diamine oxidase for the disulfide of cysteamine (*i.e.*, cystamine), and pyridoxal plus a metal ion or the enzyme cystathionine γ-lyase (γ-cystathionase; CTH), for cystine. Surprisingly, viscose dialysis tubing is also effective [17]; it is manufactured by treating cellulose with carbon disulfide and it contains residual sulfane sulfur chains unless it is exhaustively boiled in water before use. Thiols in the second group at high concentrations (10 mM) were shown to release H_2_S from fetal calf serum according to Equation (1) [18]:

Protein-S-SH + 2R-SH ⇆ H_2_S + protein-SH + R-S-S-R (1)

At lower concentrations (0.1 mM to 1 mM) these thiols do not liberate the sulfur as H_2_S but incorporate it by exchange and transport it as the persulfide (RSSH; Equation (2)). The chemistry of this reaction is discussed in detail below:

Protein-S-SH + R-SH ⇆ R-S-SH + protein-SH
(2)

**Table 2:** Physiologically compatible systems which generate sulfane sulfur from disulfides. 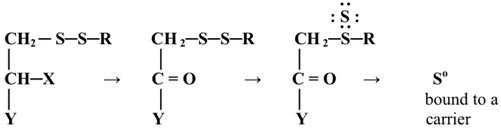

Substrate	R	X	Y	Catalyst	Refs.
Cystine	Alanine	NH_2_	COOH	Cystathionase	[19]
Cystine	Alanine	NH_2_	COOH	Pyridoxal	[20]
Cysteine-alkyl disulfides	Alkyl	NH_2_	COOH	C-S lyases	[21,22]
Cystamine	CH_2_-CH_2_-NH_2_	NH_2_	H	Diamine oxidase	[23]
Mercaptoethanol disulfide	CH_2_-CH_2_-OH	OH	H	Alcohol dehydrogenase	[17]

The stimulatory effect of sulfite on cell growth as reported by Fanger *et al*. [11] is explained by the well-known, reversible, and pH-dependent addition of S^0^ to sulfite to generate thiosulfate [24]. The sulfite ion (SO_3_^2−^) accepts S^0^ from protein carriers and acts as a low molecular weight carrier of sulfane sulfur in the form of thiosulfate (S_2_O_3_^2−^):

SO_3_^2−^ + protein-S-SH ⇆ S_2_O_3_^2−^ + protein-SH (3)

The cumulative data indicate that the growth factor is the sulfur atom (sulfane sulfur, S^0^). The sulfur-dependent murine cancer cells were found to have two genetic defects. These cells are completely lacking in the enzyme methylthioadenosine nucleoside phosphorylase (MTAP) [25] and deficient in CTH [17,26]. In direct comparison, cells containing MTAP are not dependent on the sulfur factor [25]. Subsequent to the report of the absence of MTAP in mouse cells, the enzyme was found to be absent in a large number of human cancers [27,28]. *In vivo*, these defective cells can survive by obtaining the growth factor from normal cells in the body. Isolated macrophages were found to be effective “nurse” cells for the sulfur-dependent cell lines [18].

The useful application of xenobiotic sulfur compounds (MER, TGL, and TEA) in biological systems has continued to expand. Aside from their absolute requirement in the MTAP- and CTH-defective cells, these precursors of sulfane sulfur have dramatic effects in increasing the viability, health, vigor, and proliferative capacity of many cell types *in vitro*. Today, MER, TGL, or TEA are routinely added to many *in vitro* cell systems involving immune cells, hematopoietic cells, reproductive cells, embryonic cells, and stem cells [29,30,31]. These sulfane precursor compounds are added at μM concentrations and, by integration of kinetic parameters, it can be shown that the concentration of sulfane sulfur in the media at any time is in the nanomolar (nM) range [17].

These sulfane sulfur precursors have been shown to have other effects; cystamine has been shown to have potent anti-HIV effects when the virus is grown in lymphocytes [32,33,34]. Cyst(e)amine has beneficial effects in animal models of neurodegenerative diseases (Huntington disease and Parkinson disease) and is currently in clinical trial for treating Huntington disease [35]. Members of a family of phosphorothioates of the type R-NH-CH_2_-CH_2_-S-PO_3_H_2_ have been much-studied as protectants against radiation-induced damage and chemotherapy toxicity [36]. These compounds are designated as a WR (named after the Walter Reed Army Institute of Research where they were first developed) series (e.g., WR-2721, WR-151327). These compounds are thought to act by increasing antioxidant activity such as that due to manganese superoxide dismutase [37]. There are several reports showing that MER, given orally long-term to mice, prevents the spontaneous cancers common in mice and dramatically increases longevity [38,39,40,41]. It should be noted that, although the compounds are frequently used in the thiol form, it is the disulfide which predominates in an aerobic environment.

At the time of the discovery of the beneficial effect of sulfane sulfur precursors on cells in culture, S^0^ was already known to be involved in several regulatory processes *in vitro*: activation or inactivation of a large number of enzymes, post-transcriptional modification of transfer RNA, and the biosynthesis of iron-sulfur clusters and molybdenum cofactor. The literature was reviewed in 1989 [42]. In the ensuing years, the role of sulfane sulfur in biosynthetic processes has undergone rapid development and has been reviewed [43,44] These biosynthetic processes involving sulfane sulfur involve not only the synthesis of Fe-S clusters and molybdopterin in mammals but also of biotin, thiamin, and lipoic acid in microorganisms.

## 4. Sulfane Sulfur from Garlic

Plants of the genus *Allium* are of interest because of their sulfur compounds [45]. Disrupted tissues of these plants contain several compounds which either contain sulfane sulfur (e.g., allyl disulfides and diallyltrisulfides) or generate S^0^ during simple metabolic changes that result in β elimination (e.g., alkyl-cysteine disulfides) [21]. These vegetables or the pure sulfur-containing compounds known to be present in them have been reported to have an extensive array of health-related effects. Reported beneficial effects include prevention of carcinogen-induced cancer [46,47], dementia [48] and diabetes [49]; lowering of blood cholesterol [50]; decreased plasma homocysteine levels; and prevention of atherosclerosis and heart disease [51].

“Aged garlic extract” (AGE) [52] is a product of current interest. This is a commercial product prepared by aging minced garlic in 20% ethanol at room temperature for 18 months and then removing the solids. During the aging process, the native sulfur compounds such as alliin and the odoriferous compound, allicin, are slowly converted to compounds which are not only non-odoriferous but better sources of sulfane sulfur than the compounds in the non-aged extracts. These constituents include cysteine alkyl disulfides, cysteine mercaptoallyl disulfide, diallyl disulfide, and diallyltrisulfide. Clinical trials with AGE or these pure chemicals have yielded promising but frequently conflicting results in relation to health effects (not reviewed here, but see [53]).

## 5. “Hydrogen Sulfide”

Since 1996 there have been many reports describing the effects of “hydrogen sulfide” in various biological systems [54]. The agent has been added to the systems usually as a pH-neutral water solution of NaHS. The solutions were generally used in an aerobic environment and, therefore, contained numerous sulfur species, including H_2_S, HS^−^, S^2−^, S^0^, and HS_n_S^−^ (with n varying from 1 to 8) as well as side-products from the autoxidation of H_2_S namely H_2_S(O), HS• (thiyl radical), H_2_O_2_, O_2_•, and OH• [55]. There has been no rigorous identification of the active agent in this mixture. The effects of this mixture in physiological systems have been reported to be inhibitory or stimulatory. The inhibitory effects may be due to poisoning of the cytochromes of the respiratory chain and the oxygen radicals could have other inhibitory effects. It has been pointed out by several authors that the stimulatory effects may be due to S^0^ generated by autoxidation of the sulfide [56,57,58]. This is feasible since S^0^ is active at nM to μM concentrations [17] and the H_2_S reagent is added at μM to mM concentrations [54]. Therefore, even a small degree of autoxidation would provide the active factor. This mechanism is supported by evidence that oxygen is required for the vasoactive effects of sulfide solutions in at least two systems [59,60], and is further supported by the proposed mechanism of action involving insertion of a sulfur atom into sulfhydryl groups [61], a process that can occur only with the S^0^ species and not with a sulfide species [62].

The uncertainty over the nature of the active agent in NaHS solutions might be resolved by comparing the biological effects of NaHS with those of pure sulfane sulfur-generating systems such as those listed in Table 2. Other sources of S^0^ have been found to be biologically active in the H_2_S test systems. For example, the proposed “therapeutic” compounds such as derivatives of thiophosphate and 1,2-dithiacyclopentene-3-thiones (called dithiolethiones) [63] are, in fact, sources of sulfane sulfur (not H_2_S). The sulfur in thiophosphate is clearly a sulfane sulfur and thiophosphates (e.g., Lawesson’s reagent) are used for introducing elemental sulfur atoms during organic syntheses [64]. The dithiolethiones, first developed for the vulcanization of rubber, have been extensively studied as anticarcinogenic agents where they are frequently compared to sulfur compounds obtained from garlic [65]. More recently, sulfane chains present in preparations of Na_2_S_3_ and Na_2_S_4_ have been reported to be highly active in a test system involving signaling in the brain (320-fold more active than NaHS) [66]. The fact that these sulfane chains are present in the “H_2_S” test systems supports the conclusion that the active agent is sulfane sulfur rather than hydrogen sulfide. It should be noted that the proposed agents (thiophosphates and dithiolethiones) are less appropriate as “therapeutic” agents than are mercaptoethanol disulfide and cystamine, which have already been tested in animals [29,30,31].

## 6. Properties of Sulfane Sulfur

The combined evidence from three fields of sulfur research (sulfur growth factors, sulfur compounds in garlic, and “hydrogen sulfide”) indicate that there is a form of sulfur which has remarkably wide-ranging effects in biological systems. This active form of sulfur is difficult to name and define. It has been called “zero valent sulfur”, “sulfane sulfur”, and “sulfur-bonded sulfur” but technically it can be defined as “thiosulfoxide sulfur or any sulfur atom which can tautomerize to the thiosulfoxide form”. It has six valence electrons and readily accepts two dative electrons from another sulfur atom to complete the Lewis eight electron rule [42]. The thiosulfoxide bond is weak [6] and the sulfur is easily ejected as elemental sulfur, transferred to another sulfur atom, or reduced to H_2_S by thiols. These properties apply to the sulfur in several classes of sulfur compounds:

(a) *Thiosulfoxides in the oxidation series ranging from thiosulfenic acid to thiosulfate as shown in the fourth row of Table 1.* These compounds should be considered as having two kinds of sulfur, the inner sulfur with a variable oxidation number (e.g., +4 for thiosulfate) and the outer (sulfane) sulfur with an oxidation number of zero.
(b) *Chains of sulfur atoms in which one sulfur atom can move to the thiosulfoxide position on one of the other sulfur atoms*. This includes elemental sulfur, persulfides (RSSH), polysulfides (R-S_n_-R) where n is 3 or greater, and polythionates (^−^SO_3_-S_n_-SO_3_^−^) where n is 3 or greater. Disulfides (R-S-S-R) are not in this category unless one C-S bond is activated.
(c) *Disulfides in which one sulfur is activated by a C=O, C=C, or C=N group adjacent to a C-S bond.* The activating effect of these unsaturated groups has been documented for the β-ene group in allyl disulfides [1,67] (Equation (4)), the β-keto group created *de novo* during oxidation of cystamine and mercaptoethanol disulfide as referenced in Table 2 (Equation (5)), and the β-ketimine intermediates in pyridoxal 5'-phosphate (PLP)-catalyzed reactions, such as the desulfuration of cysteine to alanine by the desulfurases discussed below (Equation (6)).
(d) *α or β-keto thiols*. The sulfur of these compounds behaves as sulfane sulfur although it cannot form a thiosufoxide. The classical example is mercptopyruvate [68]. An analogous weakening of the C-S bond is seen when alanine-3-sulfinate is transaminated to sulfinyl pyruvate during the biodegradation of cysteine [69]. For the keto group in the *β* position, the mechanism may involve keto-enol tautomerization resulting in C=C group adjacent to the C-S bond [70].

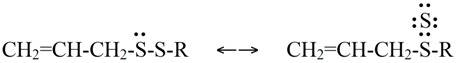
(4)

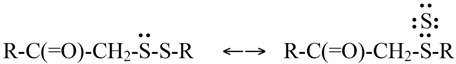
(5)

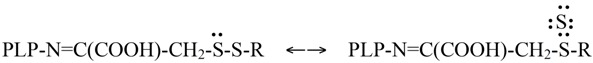
(6)

The lability of the thiosulfoxide bond provides a facile mechanism for the reversible transfer of sulfur atoms into or out of protein sulfhydryl groups (Equation (7)) and disulfides (Equation (8)):
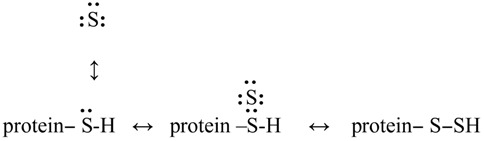
(7)

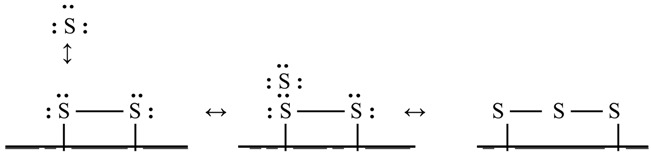
(8)


## 7. Sulfane Sulfur Transport

### 7.1. The Rhodanese Homology Domain

Sulfane sulfur does not occur in the free form (as shown schematically in Equations (7) and (8)) but is always carried on another sulfur atom. In biological systems, there is a family of carrier proteins that includes rhodanese (thiosulfate-cyanide sulfur transferase) [71], mercaptopyruvate sulfur transferase [68], CTH [72], and serum albumin [73]. The first two are “dedicated” sulfane sulfur carriers in which the sulfur atom is carried as a persulfide on a cysteine residue in a specific domain called the rhodanese homology domain (RHOD) [74]. This highly conserved domain is present in at least 500 proteins in organisms ranging from archaea to humans, including at least 47 in humans. It is present in several classes of proteins, notably the phosphatases of the CDc25 family which help to regulate the cell cycle [75].

The enzymes which incorporate S^0^ into functional molecules (MoCo, tRNA) contain the RHOD [43,44]. In molybdenum cofactor synthesis, S^0^ is converted to an intermediate thiocarboxylate, R-C(S)-O^−^, before insertion into the cofactor [9]. As stated above, MoCo occurs in the active site of sulfite oxidase [9]. A rare congenital defect in MoCo synthesis in humans results in defective sulfite oxidase and is lethal shortly after birth.

### 7.2. Non-RHOD S^0^ Binding to Proteins

Equations (7) and (8) show how sulfane sulfur can bind non-specifically to proteins. This can lead to persulfide or polysulfide groups on the cysteine of proteins (Equation (7)) or to polysulfide links between cysteine residues in proteins (Equation (8)). When CTH catalyzes the degradation of cystine, some S^0^ becomes bound to the CTH and a trisulfide structure was proposed [72]. A polysulfide link occurs in Cu-Zn superoxide dismutase [76] and has been identified in other proteins [77] particularly in antibodies [78]. In a recent paper, Ida *et al.* showed that polysulfide groups on thiols and proteins can be stabilized by bromobimane derivatization and that the derivatives can be separated and identified by GC-MS—a technique which they called “polysulfidomics” [58]. Polysulfide groups were found on a surprisingly diverse set of proteins.

Polysulfides are frequently found in proteins produced by recombinant technology and this may explain (in part) the unexpected inactivity or altered reactivity of many enzymes produced by this technology. Caution must be used in interpreting these findings in proteins prepared by dialysis because the sulfane sulfur can be inadvertently introduced from the viscose dialysis tubing.

It is likely that this type of sulfur binding occurs *in vitro* in enzymes reported to be affected by S^0^ but which are not known to contain the active RHOD [42]. This type of bonding may occur also with cyclin-dependent protein kinase p34cdcK which is inhibited by the S^0^ source diallyldisulfide [79] and with the protein tyrosine phosphatase PTP1B which is inactivated by solutions of NaHS [80].

## 8. Sulfane Sulfur Generation

Following is a list of biochemical systems that can generate sulfane sulfur. In general, the systems involve the generation of a group which labilizes a C-S bond. The activating groups (C=O, C=N, or C=C) are known to delocalize electrons in other systems such as in aldol condensation, PLP-catalyzed reactions, and allylic rearrangement, respectively.

### 8.1. Cysteine Deamination (Generation of a C=O Group α to a C-S Bond)

Deamination of cysteine to β-mercaptopyruvate (MP) can occur by transamination [68] or by oxidative deamination catalyzed by L-amino acid oxidase [81]. MP was the first described example of a biological compound containing a carbonyl-activated sulfur atom [68]. *In vivo*, there is a specific RHOD-containing carrier protein which accepts the S^0^ from mercaptopyruvate resulting on the formation of pyruvate. When the deamination is carried out *in vitro* with supraphysiological concentrations of cysteine, the residual cysteine has considerable reducing capacity and much of the sulfur is reduced from MP as H_2_S by the excess cysteine [81]. The oxidation of the compounds shown in Table 2 are additional examples in which a carbonyl group is created adjacent to a C-S bond, thereby labilizing it.

### 8.2. Homocysteine Deamination (Generation of a C=O or C=N Group β to a C-S Bond)

l-Homocysteine is a substrate for transamination by glutamine transaminase K [82] and oxidative deamination [81,82]. With bacterial or snake venom l-amino acid oxidase, the keto acid is formed along with some H_2_S. The release of the sulfur was attributed to the labilizing effect of the keto or imino group (possibly involving keto-enol tautomerization as studied by Nicolet [70]), The reducing effect of the excess thiol substrate would cause release of some sulfur as H_2_S. In addition, d-homocysteine is a substrate of mammalian d-amino acid oxidase [82 and references therein].

Given the biomedical importance of homocyst(e)ine in health and disease the possibility that homocyst(e)ine may be a source of sulfane sulfur by mechanisms similar to those outlined above needs to be further investigated.

### 8.3. Cysteine Desulfurases (C=N in the α Position)

Cysteine desulfurases remove the sulfur from cysteine, thereby generating alanine. These are PLP-containing enzymes in which the formation of the ketimine group adjacent to the C-S bond is sufficient to release the sulfur atom without net removal of the amino group [83]. These desulfurases provide the sulfur for the synthesis of iron sulfur clusters and MoCo and for the modification of transfer RNA in all species thus far investigated, including humans [84], and for the synthesis of lipoic acid, biotin, and thiamin in bacteria [85].

### 8.4. Cysteine S-conjugate Lyases (C-S Lyases) (β Elimination)

Many PLP-containing enzymes catalyze β-elimination reactions with cysteine *S*-conjugates, generating ammonia, pyruvate, and a sulfur-containing fragment [21,22,86]. In many cases, the β-elimination reaction is biologically important (e.g., the reaction catalyzed by cystathionine β-lyase). However, in other cases (particularly when the amino acid substrate contains a good leaving group in the β position), the PLP-enzyme is “coerced” into catalyzing a non-physiological β-elimination reaction. For reviews see [21,86]. When the substrate is a sulfide, the sulfur product is a thiol (Equation (9)) and when the substrate is a disulfide, the product is a persulfide (Equation (10)):

cy-S-R + H_2_O → NH_3_ + pyruvate + R-SH (9)

cy-S-S-R + H_2_O → NH_3_ + pyruvate + R-S-SH (10)

Cysteine *S*-conjugate β-lyase activity is associated with a surprisingly diverse set of PLP-containing enzymes (at least nineteen) including kynurinase, several aminotransferases and CTH in mammals [86], and several amino acid decarboxlyases in a variety of microorganisms [87].

The R group depicted in Equations (9) and (10) may be a member of a large spectrum of groups, e.g., alkyl, halogenated alkyl, halogenated alkene, halogenated alkyne, aryl, halogenated aryl, benzothiazole, cysteine (reviewed in [21,22,86]). Reactions shown in Equation (10) lead to sulfane sulfur directly in the form of a persulfide whereas reactions shown in Equation (9) do so indirectly. Thus, in Equation (9) when R is a small alkyl group, an alkyl thiol is released and, *in vivo*, this thiol enters disulfide exchange with cystine giving rise to the mixed disulfide cy-S-S-R which then enters a new cycle of the C-S lyase system according to Equation (10). Methane thiol arising from the catabolism of methionine [88] and *S*-alkyl cysteines present in plants of the *Brassica* family could theoretically be metabolized to a compound (methane thiol persulfide; methyldisulfane) that contains sulfane sulfur by this mechanism (see below).

### 8.5. Cystathionine γ-Lyase (CTH)

CTH is a special example of a C-S lyase and the prototype of these enzymes. Since it is widely cited as a major source of S^0^ and H_2_S [54,66], it merits special comment. With its nominal substrate, l-cystathionine, the enzyme catalyzes a γ-elimination reaction yielding α-ketobutyrate (Equation (11)). Under in vitro test conditions, CTH has the ability to catalyze β-elimination reactions with several disulfides; cystine (Equation (12)) [19], various alkyl cysteine disulfides (Equation (10)) [89], and cysteine-3-mercaptolactate disulfide (cy-S-S-lactate) (Equation (13)) [90], in each case yielding a persulfide. Cy-S-S-lactate occurs in the blood in the congenital defect in mercaptopyruvate sulfur transferase and is associated with mental retardation and other defects [91]:

cy-S-hcy + H_2_O → NH_3_ + α-ketobutyrate + cysteine (11)

cy-S-S-cy + H_2_O → NH_3_ + pyruvate + cy-S-SH (12)

cy-S-S-lactate + H_2_O → NH_3_ + pyruvate + lactate-S-SH (13)

Because of the low degree of discrimination of CTH for substrates, it is likely that the mammalian enzyme can also degrade cysteine-homocysteine disulfide (cy-S-S-hcy). Two bacterial homologues are known to use this mixed disulfide as a substrate [92,93]. The mixed disulfide, cy-S-S-hcy, is present in normal human plasma at a concentration of ~3 μM but may reach a concentration of 30 μM in the plasma of patients with hyperhomocysteinemia [94,95,96]. With the mammalian CTH, the reaction could be either a β-elimination reaction (Equation (14)) or a γ-elimination reaction (Equation (15)):

cy-S-S-hcy + H_2_O → NH_3_ + pyruvate + hcy-S-SH (14)

cy-S-S-hcy + H_2_O → NH_3_ + ketobutyrate + cy-S-SH (15)

In both cases the eliminated fragment is a persulfide. The generation of excessive (toxic) amounts of S^0^ from cy-S-S-hcy may account for some of the pathology seen in hyperhomocysteinemia and should be further investigated. There is some evidence that CTH activity is regulated so as to maintain a constant level of S^0^ availability. In strains of the fungus, *Aspergillus nidulans*, defective in enzymes of cysteine synthesis and having low cysteine availability, the enzymes rhodanese and CTH are increased in activity interpreted as a homeostatic mechanism for maintaining the S^0^ level [97].

### 8.6. Allyl Disufides (C=C Group in the α Position)

Alllyl disulfides undergo spontaneous rearrangement [1,67]. If the reaction is carried out in the presence of triphenylphosphine, one sulfur atom is removed, proving that the mechanism involves a thiosulfoxide intermediate. (For details of this complex rearrangement, see [1,67]). Because of this property, the allyl sulfur compounds which occur in garlic (e.g., diallyl disulfide) contain sulfur which is reactive as sulfane sulfur.

### 8.7. The Polyamine Pathway 

The evidence from MTAP-deficient mouse cells indicates that this pathway is important for S^0^ generation, at least in mice. In this pathway, the sulfur of methionine is first converted to methyl mercaptan via the following sequence: methionine → *S*-adenosylmethionine → decarboxy-*S*-adenosylmethionine → 5'-methylthioadenosine → 5-methylthioribose-1-phosphate → 2-oxo-4-methylthioribose →→→ 3-methylthio-propionyl coenzyme A → methyl mercaptan. (For the detailed pathway, the enzymes involved, and numerous references see [98]). The methyl mercaptan then forms a mixed disulfide with cysteine by disulfide exchange with cystine and the mixed disulfide is a substrate for the C-S lyases according to the reaction shown in Equation (10). In humans, this pathway may be important in the embryo where polyamine synthesis is rapid [99]. The C-S lyase in the embryo is not CTH since that enzyme is absent in the embryo [100,101], but there seems to be a consensus that CTH is an important source of S^0^ after the first year of life when this enzyme is highly expressed in human liver.

## 9. The “Antioxidant” Properties Attributed to Sulfur Compounds

Numerous publications attribute “antioxidant” properties to sulfur compounds even when the compounds have no reducing or other radical-quenching groups [102]. In most cases, there is no obvious antioxidant mechanism. The intended meaning seems to be that the cells or tissues are more healthy and vigorous in the presence of the sulfur compounds and, in some cases, there is an increase in reductants (such as glutathione) chemically unrelated to the added sulfur compound. One such compound is S-allylcysteine for which various mechanisms have been proposed [102]. Other examples are the xenobiotic disulfides of MER, TGL, and TEA which, as disulfides, have no reducing ability but have potent effects in promoting the replication and health of many cell types. Indeed, they are ineffective in cell cultures when in the reduced (thiol) form [17]. However, the “antioxidant” properties of these sulfur compounds is explainable by their ability to give rise to sulfane sulfur, which then exerts antioxidant effects through the following mechanisms:

### 9.1. Superoxide Dismutase Requires S^0^

Copper-zinc superoxide dismutase (CuZn SOD) has a sulfane sulfur bridge between Cys111 residues of the two units of the dimeric form; the number of sulfur atoms in the sulfane sulfur bridge varies depending on the method of purification, with numbers as high as 7 [76]. The stability of the enzyme is enhanced by the presence of these sulfur atoms [76] and CuZn SOD activity *in vitro* is increased in the presence of aerobic (and, hence, S^0^-containing) solutions of NaHS [103]. Therefore, sulfuration of CuZn SOD may contribute to the anti-oxidant effects of sulfane sulfur precursors. Since H_2_O_2_ is the product of SOD, the increased production of H_2_O_2_ in the cancer cells treated with garlic-derived sulfur compounds [104] may be attributable to the increased activity of this enzyme as a result of addition of sulfane sulfur to the enzyme. Thus, Iciek *et al*. studied the effects of various sulfur-containing compounds derived from garlic on the production of H_2_O_2_ in HepG2 cells. Diallyl trisulfide, an S^0^-containing compound that occurs in garlic, was found to be especially effective in stimulating H_2_O_2_ production [104].

### 9.2. The Sulfur in the Iron-sulfur Clusters Originates as S^0^

Iron sulfur clusters have the ability to process electrons near the lower limit of the physiological redox range. The clusters contain variable numbers of iron and sulfur atoms with each variation adapted to specific functions [105]. Although the sulfur in these clusters is in the sulfide form, it originates as sulfane sulfur (persulfide) which undergoes reduction during the synthesis of the clusters [42,105]. The functions of iron sulfur clusters in bacteria are extremely diverse but in higher animals the functions are mainly in the electron transport chain of oxidative phosphorylation (complexes I, II, and III), in steroid synthesis (adrenodoxin), and in the generation of deoxyadenosyl radical (ado•) by an enzyme called MoaA. The ado• radical is formed when one electron is transferred from the iron of an iron sulfur cluster to S-adenosylmethionine resulting in the release of methionine:

S-adenosyl methionine + e → methionine + ado•
(16)

This radical has numerous functions in bacteria and archaea [106] but in mammals its main function is in the biosynthesis of molybdenum cofactor (MoCo) from guanosine triphosphate [107]. As stated above, MoCo functions in disposing of the end-product of sulfur metabolism, sulfite ion, as well as purine catabolites (xanthine) in animals [9]. Therefore, through iron sulfur clusters, sulfane sulfur contributes indirectly to the redox regulation as well as the disposal of the end product of sulfur metabolism.

### 9.3. The Reducing Capacity Is Increased in S^0^-stimulated Cells

As stated above, sulfane sulfur greatly enhances the vigor and health of cells. This increased vigor could indirectly increase the cellular content of oxygen-defensive factors such as glutathione, catalase, and superoxide dismutase. Numerous reports have described an increase in cellular glutathione in the presence of sulfur compounds related to sulfane sulfur [29,30,31,45,54]. The presence of air-exposed solutions of NaHS has been shown to increase the expression of Mn-SOD in ischemia-stressed cardiomyocytes as well as the *in vitro* activity of CuZn SOD [103].

### 9.4. Glutathione Persulfide (G-S-SH) Is a Powerful Reductant

In 1971, Massey *et al.* reported the remarkable finding that S^0^ impurities in commercial GSSG samples catalyze the rapid reduction of cytochrome *c* by the GSH [108]. GSH alone or GSH in the presence of pure GSSG (freed of S^0^) did not reduce cytochrome *c*. The impurity in GSSG was identified as the trisufide, GSSSG, and it could be replaced by cystine trisulfide, cy-S-S-S-cy, or elemental sulfur. The sulfane sulfur was catalytic in this process; only the GSH was consumed in the reduction of cytochrome *c*. The authors concluded that the S^0^ was introduced into this system by GSSSG impurities in the GSSG and that the active agent in cytochrome *c* reduction was the persulfide GSSH, *i.e.*, that the persulfide is a much more effective reducing agent than is GSH.

Prütz described the application of this reduction system to resazurin, a phenoxazine dye which turns red and highly fluorescent when reduced and which is used to indicate viability of cells [109]. He showed that sources of S^0^ increased the rate of reduction of resazurin by GSH; irradiated cystamine increased the rate by 30-fold and tetrathionate increased the rate by 60-fold (tetrathionate gives rise to S^0^ by partial reduction to thiosulfate by GSH). The remarkable increase in reducing capacity of GSSH relative to GSH is not fully explored, but it appears likely that the persulfide tautomerizes to the thiosulfoxide form and that the thiosulfoxide donates electrons with great facility:

2 GSSH → 2 GS(S)H → 2e + 2H^+^ + GSS-SSG (17)

These findings were unappreciated for many years. However, a recent publication [58] seems to have re-discovered the effect first reported by Massey *et al*. (without citing the earlier work). In this study GSH (supported by NADPH and glutathione reductase) destroyed ~5% of added H_2_O_2_ in 30 min whereas the same system containing persulfide destroyed 100% of the H_2_O_2_ in 30 min. The polysulfidomic analysis revealed that about 10% of glutathione in tissue occurs in the GSSH form [58].

In summary, the effects of many sulfur compounds which have been interpreted as due to anti-oxidant properties are probably indirect and mediated through the sulfane sulfur generated from these compounds.

## 10. Sulfur Compounds and Elemental Sulfur in Plant Defense

There is growing evidence that the ability of plants to defend against virus and fungus infections is related to the availability of sulfate from which plants make all of their sulfur compounds. This has led to the consideration of including sulfate in agricultural fertilizer. Virus or fungus-infected plants have been shown to have increased levels of thiol compounds (cysteine, GSH), thiocyanates (called glucosinolates), and thiazole compounds (called phytoelexins) all of which can have anti-microbial properties; and to excrete H_2_S gas. There is a coincident synthesis of cysteine-rich proteins called defensins, the function of which is not known. These are a family of small peptides each having 8 cysteine residues in a total 18 to 45 amino acids [110]. 

A remarkable finding is the accumulation of elemental sulfur (S_8_) in xylem tissue of certain plants infected with appropriate pathogenic fungi. This has been demonstrated in tomato, cocoa, cotton, tobacco, and French bean challenged with appropriate fungi but did not occur with strawberry or maize at least with the fungi tested. Sulfur accumulation was faster and greater in genotypes recognized as “resistant” to the fungus than in genotypes known to be “susceptible”. Sulfur was not detected in un-infected control plants. In *in vitro* testing, the fungi in question showed growth inhibition by elemental sulfur. These findings suggest that there has been a remarkable natural selection for and synthesis of an effective anti-fungal agent by certain plants. This reflects the centuries-old tradition of man in using powdered sulfur as an anti-fungal agent in agronomy. The excretion of H_2_S in infected plants (mentioned above) may be a result of the reduction of some of the elemental sulfur. This emerging subject has been reviewed [111,112].

## 11. Is There a Selenium Analog of S^0^?

There is a rapidly-growing literature on the beneficial effects of selenium compounds (selenite, selenate, selenocysteine, selenomethionine) on heart disease, cancer prevention, immunity, diabetes, and dementia [113]. Therefore, it is logical to ask whether there is a form of selenium analogous to S^0^. 

Selenium was, for a long time, the neglected congener of sulfur. However, its unusual biochemistry has been brilliantly advanced in recent years in the laboratory of Thressa Stadtman [114]. In mammals, selenocysteine (cysteine in which the S is replaced by Se) is a constituent amino acid in several mammalian enzymes: glutathione peroxidase, thioredoxin reductase, iodothyronine deiodinase, and methionine sulfoxide reductase. Selenium is activated as selenophosphate for incorporation into selenocysteine which has its own unique tRNA (tRNA^Sec^) and codon for incorporation into proteins. The unique codon is UGA, which is normally a stop codon, but as a result of the special environment within the message binds instead selenocysteine-tRNA^Sec^. 

The role of covalently-linked selenium in selenocysteine-containing enzymes is clear but there is much less information on Se^0^ (the analog of S^0^). Se^0^ is referred to as “perseleno selenium” but that name does not represent all of the possible structures which include R-Se-SeH, R-S-SeH, and R-Se-SH. The “triselenide” of glutathione is readily formed *in vitro* from selenite (selenium dioxide in water) by the reaction shown in Equation (18) [115]. Stadtman *et al.* showed that the selenium in that derivative can be bound and carried by rhodanese *in vitro* [116]. There is a protein, selenoprotein P, which has 10 selenocysteine residues and is thought to act as a transporter of the amino acid, selenocysteine, for example from the liver to the brain [117]:

4 G-SH + SeO_2_ → G-S-Se-S-G + G-S-S-G + 2 H_2_O (18)

The selenium field is seriously overshadowed by the potential toxicity of selenium. Its history in nutrition began 80 years ago when it was noted that livestock were poisoned after ingesting selenium-rich plants in Western United States (reviewed in [118]). There have been reports of clusters of selenium poisoning in humans resulting from dietary supplements containing excessive amounts of selenium compounds as described in reports from the CDC in 2010 [118] and from others [119]. Selenium poisoning was epidemic in a district of China that has selenium-rich soil [120], and there is an incident of the death of 20 polo horses after injection with a stimulant containing high amounts of selenium [121]. (see [121] for precise details of lethal doses of selenium). The exact mechanism of selenium toxicity has not been determined giving rise to further uncertainty about its clinical use. For example, the possible role of selenium in cancer prevention is tempered by the possibility that it could damage DNA and cause cancer [122]. Selenium supplements have been shown to decrease dementia symptoms in a mouse model of Alzheimer’s disease [123] suggesting clinical potential. However, there is the contravening finding that selenoprotein P is found in abundance in the plaque of Alzheimer’s disease brains [124]. This raises the disturbing possibility that this protein may contribute to the disease process and indeed, memory loss is a symptom of selenium poisoning in humans.

Does perseleno selenium have biological roles analogous to S^0^? It appears that it can be generated *in vivo*, that it may be carried on sulfurtransferases, and that it has some similar functions such as the selenation of certain tRNAs. However, there is another (and probably more important) similarity between S^0^ and Se^0^ which may explain the observed effects of selenium and may even explain its toxicity. As outlined above, the persulfide of glutathione, GSSH, has much greater reducing capacity than does GSH in the systems tested [108,109]. In 1993, Levander *et al.* showed that selenite could replace S^0^ in producing this effect in the cytochrome *c* system [125] and in 1994 Prütz tested selenite in the resazurin system [109]. The striking result was that GSH reduced resazurin 50 times faster with selenite as a source of Se than it did with tetrathionate as a source of S^0^. The rate with selenite was ~3000 times faster than the rate with GSH alone. The ratio of selenite to GSH was 1:100. It was concluded that the active reductant is the perselenide of GSH, GSSeH, formed by two reactions; the selenite is first reduced to GS-Se-SG by GSH according the Equation (18) and the GS-Se-SG then gives rise to GS-SeH through an exchange reaction with another GSH according to Equation (19):

GS-Se-SG + GSH ⇆ GS-SeH + GSSG
(19)

The effect of selenium in facilitating reduction reactions is not restricted to the systems described above; GSH can be replaced by other thiols (e.g., cysteine, mercaptoethanol) and the effect occurs in other redox systems. Thus, Rhead and Schrauzer showed that the reduction of methylene blue by mercaptoethanol is increased 20-fold by the presence of trace amounts of selenite [126]. However, the effect *in vivo* is likely to apply mainly to GSH because of its high concentration in cells (1 to 10 mM) and the role of GSH in determining the redox status in cells. This remarkable reducing property of GSSeH needs to be explored in more detail to determine whether it may explain some or all of the observed beneficial effects of selenium compounds in biological systems. It may even account for the toxicity of selenium by creating an over-reducing redox environment.

## 12. Conclusions

Rapidly accumulating data indicate that sulfane sulfur has important functions in cells. The broad diversity of effects suggests that its functions are general and not specific to any tissue or any process. Moreover, it should not be called a “signaling agent” since there is no evidence that it acts in a controlled rise and fall pattern (as with neurotransmitters or hormones). Rather it appears to be an essential factor that must be available at low and constant concentration. Its overall effect is to keep all cells in an optimum state of health with regard to viability, vigor, longevity, and proliferative capacity. There are several mechanisms by which S^0^ could have this effect in mammals. These include maintenance of sulfur-containing cofactors (MoCo, Fe-S clusters), the control of protein synthesis via modification of tRNA, the regulation of the activities of enzymes, and the maintenance of the reducing capacity of cells.
